# Evaluation of Skin Permeation and Analgesic Activity Effects of Carbopol Lornoxicam Topical Gels Containing Penetration Enhancer

**DOI:** 10.1155/2014/127495

**Published:** 2014-06-19

**Authors:** Saleh A. Al-Suwayeh, Ehab I. Taha, Fahad M. Al-Qahtani, Mahrous O. Ahmed, Mohamed M. Badran

**Affiliations:** Department of Pharmaceutics, College of Pharmacy, King Saud University, P.O. Box 2457, Riyadh 11451, Saudi Arabia

## Abstract

The current study was designed to develop a topical gel formulation for improved skin penetration of lornoxicam (LOR) for enhancement of its analgesic activity. Moreover, the effect of different penetration enhancers on LOR was studied. The LOR gel formulations were prepared by using hydroxylpropyl methylcellulose (HPMC) and carbopol. The carbopol gels in presence of propylene glycol (PG) and ethanol were developed. The formulated gels were characterized for pH, viscosity, and LOR release using Franz diffusion cells. Also, *in vitro* skin permeation of LOR was conducted. The effect of hydroxypropyl *β*-cyclodextrin (HP *β*-CD), beta-cyclodextrin (*β*-CD), Tween 80, and oleic acid on LOR permeation was evaluated. The optimized LOR gel formulation (LORF8) showed the highest flux (14.31 *μ*g/cm^2^/h) with ER of 18.34 when compared to LORF3. Incorporation of PG and HP *β*-CD in gel formulation (LORF8) enhanced the permeation of LOR significantly. It was observed that LORF3 and LORF8 show similar analgesic activity compared to marketed LOR injection (Xefo). This work shows that LOR can be formulated into carbopol gel in presence of PG and HP *β*-CD and may be promising in enhancing permeation.

## 1. Introduction

Nonsteroidal anti-inflammatory drugs (NSAIDs) have analgesic and antipyretic properties [1]. Although oral NSAIDs are effective in the treatment of a variety of acute and chronic pain conditions, their use may be associated with serious systemic adverse effects, particularly gastrointestinal disorders [2]. Transdermal delivery of NSAIDs proved to be a convenient route of administration for a variety of clinical indications [3]. In addition, using of gel as a delivery system can increase the residence time of drugs on the skin and provide a faster release of drug substance [4, 5]. Extensive preformulation studies are generally necessary in order to optimize both drug release from the topical vehicle and skin permeation. Lornoxicam (LOR) is an NSAID of the oxicam class with analgesic, anti-inflammatory, and antipyretic properties. It is a potent inhibitor of both COX-1 and COX-2 enzymes. LOR exhibits a short plasma elimination with half-life (3–5 hr). LOR is characterized by lipophilic nature with a poor solubility in the acidic media of the stomach which gives local toxicity on the stomach [6, 7]. LOR has a molecular weight of 371.8, partition coefficient of 1.7, and dose of 4 to 8 mg [6], and it is available only in tablet and parenteral forms. Therefore, LOR could be considered as a good candidate for topical application. In the previous literatures, transdermal films, patches, and topical lotion for LOR were developed [8–10]. There are no reports on LOR loaded to gels containing penetration enhancers.

In the development of transdermal formulations, the selection of vehicle leads to improving the transdermal delivery [11]. It is important to optimize the topical formulations to get appropriate permeation of the drug through the skin. Hydroxypropyl methylcellulose (HPMC) has been stated to be effective in enhancing the drug permeation through human skin [12, 13]. Hence, HPMC was examined for its permeation enhancement ability in this research work. In addition, carpobol is a polymer of acrylic acid cross-linked with polyalkenyl ethers or divinyl glycol [14, 15]. It was reported that there is no skin irritation after topical application of gels containing PC [16]. It was stated that CP has hydrophilic properties and cross-linked condition, therefore CP is considered as a potential candidate for the use as a gel for topical application. The majority of the drugs do not appear to penetrate the skin at a rate sufficient enough for therapeutic efficacy because of the barrier function of the stratum corneum (SC). There were many attempts to increase the drugs flux through SC by the use of suitable penetration enhancers. Penetration enhancers exert their effect mainly by altering the nature of SC either by fluidizing its intracellular lipids and hence reducing the diffusion resistance or by disrupting the order of lipid structure and increasing the partitioning of the drug into SC from the vehicle [17]. Therefore, the objective of this study was to develop and optimize the permeation of LOR from gel formulations containing HPMC or CP in presence of PG and ethanol, which were used as penetration enhancers. Furthermore, pH, spreadability, viscosity, and* in vitro* drug release were investigated. The results were evaluated to expect the appropriate formulation which was further used for its topical delivery. Also the effect of other penetration enhancers like HP *β*-CD, beta-*β*-CD, Tween 80, and oleic acid was investigated for the transdermal diffusion of LOR through rabbit skin. Likewise, the* in vivo* analgesic activity of optimized LOR gel was studied.

## 2. Materials and Methods

### 2.1. Materials

Lornoxicam (LOR) was purchased from Beta Pharma (New Jersey, USA). Hydroxypropyl methylcellulose, triethanolamine, and propylene glycol (HPMC 4000) were purchased from Fluka chemical (Switzerland). Carbopol 974, Tween 80, and methylparaben were purchased from BDH Chemicals (LTD, UK). Potassium dihydrogen orthophosphate, ethanol, and oleic acid were purchased from Sigma Chemical Company (USA). *β*-cyclodextrin (*β*-CD) was purchased from Acros Organics (New Jersey, USA), and hydroxypropyl *β*-cyclodextrin (HP *β*-CD) was purchased from Fluka chemika, UK. LOR lyophilized vial which contains 8 mg/2 mL was also used as a reference (Xefo injectable, Nycomed). All organic solvents were of analytical grade.

### 2.2. Determination of LOR Solubility and Partition Coefficient

Excess amount of LOR powder was added in a conical flask containing 10 mL distilled water, phosphate buffer (PB pH 7.4), and PB containing various concentrations of either *β*-CD or HP *β*-CD. The suspensions were stirred at 32°C on a water bath shaker for 72 h. An aliquot was withdrawn and filtrated through 0.45 *μ*m Millipore filter. The samples were diluted with suitable solvent and analyzed by a UV-spectrophotometer at 376 nm. The concentration of LOR was then determined in triplicate.

To determine the partition coefficient, PB (pH 7.4) was used as water phase. The partition coefficient was determined using the shake flask method by dissolving known concentration of LOR in 20 mL of 50 : 50 octanol and PB mixture in a conical flask. The flask was agitated for 2 h at ambient temperature and then allowed to stand for 2 h in order to separate the layers completely. The aqueous phase was separated from the oil phase using separation funnel. The amount of LOR in each layer was measured using UV-spectrophotometer at 376 nm and partition coefficient was calculated. Partition coefficient of LOR was also determined in the presence of various concentrations of *β*-CD and HP *β*-CD in PB by the same procedures.

### 2.3. Preparation of LOR Gels

The exact amount of HPMC was dispersed in warm water with continuous stirring to form gel. The drug/triethanolamine/methylparaben mixture was prepared and the final volume was adjusted by addition of water. To prepare the carbopol gel, it was dispersed in distilled water. The carbopol dispersion was kept in the dark overnight to allow for the complete swelling. Then the amount of LOR and triethanolamine was dissolved in the specified quantity solution of methylparaben. This solution of the drug was added slowly in the aqueous dispersion of polymer to get homogeneous dispersion [15]. In formulations containing penetration enhancer, PG and/or ethanol quantities were added to the drug mixture before addition of carbopol. The composition of gel formulations is given in Table 1.

### 2.4. *In Vitro* Characterization

#### 2.4.1. pH Evaluation

The exact amount of LOR gels was weighed in a 25 mL volumetric flask and then volume was made up with double distilled water to 25 mL. The pH of the gel was measured using a digital pH meter (Seven Easy pH meter, Switzerland) by getting it in contact to equilibrate it for 1 min. The study was achieved to check the neutralization of different gels. pH evaluation was carried out in triplicate for all formulations.

#### 2.4.2. Spreadability

The spreadability of the formulated gels was measured by spreading of 0.5 g of the gel on a circle of 2 cm diameter premarked on a glass plate and then a second glass plate was employed. Half kilogram of weight was permitted to rest on the upper glass plate for 5 min [18]. The diameter of the circle after spreading of the gels was determined (*n* = 3). The following equation was used to determine the percent spread:

(1) $$\begin{array}{c} \%\text{spread}\;\text{by}\;\text{area}=\frac{A_{2}}{A_{1}}\times 100, \end{array}$$

where *A*_1_ = 2 cm and *A*_2_ = final area after spreading.

#### 2.4.3. Viscosity Measurements

The viscosity measurements of LOR gels were determined at 25°C using a Brookfield Viscometer (Model DV-E, Middleboro, MA, USA) and plate rheometer with spindle 15/21. A typical run comprised angular velocity from 0.5 to 100 rpm. All viscosity measurements were performed in triplicate.

#### 2.4.4. Differential Scanning Calorimetry (DSC)

Thermal analysis was used to elucidate any interactions between LOR and investigated polymers. DSC was carried out using Shimadzu, DSC 60 thermal analyzer with a liquid nitrogen cooling accessory. The analysis was performed under purge of dry nitrogen gas (40 mL min^−1^). A sample of 2–5 mg was placed in an aluminum crucible cell and was firmly crimped with the lid to provide an adequate seal. The samples were heated from ambient temperature to 300°C at a preprogrammed heating rate of 10°C min^−1^. All samples were analyzed in the same manner.

### 2.5. *In Vitro* LOR Release

*In vitro* release study of LOR from gels (LORF1-LORF5) was performed by using 0.5 grams of each gel formulation. The amount of gel was accurately weighed and placed on a cellophane membrane (MWCO 12–14,000) previously immersed in phosphate buffer of pH 7.4. The loaded membrane was mounted on the Franz diffusion cell with a diffusional area of 1.76 cm^2^. The receptor phase contained 12 mL of phosphate buffer. The buffer solution temperature was maintained at 37°C ± 0.5 with constant stirring. Accurate samples (1 mL) were withdrawn at time intervals 0.25, 0.5, 0.75, 1, 1.5, 2, 3, 4, 5, and 6 hr. The volume of each withdrawn sample was replaced by the same volume of same dissolution medium maintained at the same temperature to keep constant volume. The released amount of LOR was determined using HPLC method after appropriate dilution.

### 2.6. Skin Irritation Studies

After explaining the research protocol with possible side effects, the volunteers were asked to sign consent forms. The study was ethically approved by the Academic Committee of the Department of Pharmaceutics, College of Pharmacy, King Saud University.

The gel formulation of LOR that gave the highest release was selected for testing skin irritation. It was carried out on five human volunteers. A half gram of LORF3 was applied in the hand of each volunteer for 24 hours. After removal of gel, the resultant skin effects were examined for the sign of erythema or itching. The effects were classified into 5 scores depending on the degree of erythema as follows: 0 (no erythema), 1 (slight erythema-light pink), 2 (moderate erythema-dark pink), 3 (moderate to severe erythema-light red), and 4 (severe erythema-extreme redness).

### 2.7. *Ex Vivo* Skin Permeation Studies

#### 2.7.1. Preparation of Skin Membrane

Male white New Zealand rabbit was sacrificed, and the dorsal skin was excised. Hairs were removed using electric clipper; subcutaneous tissues were surgically removed without damage to the skin. The skin samples were wrapped in aluminum foil after washing by isotonic phosphate buffer (IPB) and stored in a deep freezer at −20°C until further experiment. All animals' studies were approved by the Institutional Animal Care and Use Committee. The study was conducted in accordance with the NIH Guide for the Care and Use of Laboratory Animals.

#### 2.7.2. Skin Permeation Studies

In this study, the effect of 5% penetration enhancers on the permeation of LOR through rabbit skin from LORF3 was investigated. The used penetration enhancers are Tween 80 (LORF6), oleic acid (LORF7), HP *β*-CD (LORF8), and *β*-CD (LORF9).

To obtain different skin permeation profiles, full-thickness rabbit skin mounted to Franz diffusion cells (Logan Instruments, NJ, USA) was used. Appropriate skin parts were inserted between the donor and the receptor fluid of the Franz diffusion cells with the SC facing upward into the donor compartment and the dermal side of the skin allowed to contact with receptor fluid. The Franz diffusion cell has a diffusional area of 1.76 cm^2^ and the receptor phase contained 12 mL of phosphate buffer, pH 7.4, which was stirred at 300 rpm. The receptor fluid was thermostated at 37 ± 1°C to provide a skin surface temperature at 32 ± 1°C.

A half gram of LOR gels was applied to the donor under occlusive condition to prevent evaporation. After application of the gels, 1 mL was withdrawn for the receptor fluid at different time interval and replaced with fresh and previously warmed phosphate buffer solution. The concentration of permeated drug was determined by HPLC method and the cumulative amount of drug permeated was calculated as well as the percutaneous permeability parameters.

### 2.8. HPLC Analysis

LOR was determined using a reverse-HPLC method with minor modification [8]. The HPLC system (Schimadzu, Japan) equipped with SPD-10AV UV visible variable wavelength detector, constant flow pump, LC-10AD (Schimadzu, Corporation, Koyoto, Japan), and Rheodyne injector (Model 70, Rheodyne Inc., Catati, CA, USA) was used. The HPLC system was monitored by computing integrator C-R4A chromatopac (Schimadzu). LOR was analyzed using mobile phase that consisted of aqueous phosphate buffer and methanol in a ratio of 6 : 4 (v/v) and pH was adjusted to 7 by using 1 M sodium hydroxide. The mobile phase was filtered through 0.22 *μ*m Millipore filter under vacuum and degassed before being used. The mobile phase flowed over a reversed-phase Nova-Pak C18 column (150 × 3.9 mm) (Restek Corporation, Bellefonte, PA, USA) at a rate of 1 ml/min. The injection volume of each LOR sample was 20 *μ*L and was detected by the UV detector at 280 nm. All the operations were carried out at room temperature. Typical chromatogram of LOR gave retention time of 6.01 minutes.

### 2.9. *In Vivo* Analgesic Activity

Adult male albino mice (30–32 g) were used for this experiment. The animals (one per cage) were maintained under standard laboratory conditions (light period of 12 hr/day and temperature maintained at 25°C ± 2°C), with free access to food and water. A washing period of 5–7 days between dosages was applied. All studies were approved by the Institutional Animal Care and Use Committee and were conducted in accordance with the NIH Guide for the Care and Use of Laboratory Animals. The analgesic activity of LOR was studied by the hot plate analgesic method. The method is described by Shetty and Anika [19] and modified by Franzotti et al. [20]. Each mouse was placed on a hot plate (MOD 39D Hot meter, Columbus, USA) maintained at 55 ± 1°C [21] and the pain reaction time (PRT) or latency period (the time taken for the mice to react to the pain stimulus) was recorded. The response to pain stimulus includes jumping, raising, or licking of the hind foot. It was reported that the effective intraperitoneal (ip) dose of LOR is 1.3 mg/kg in rats [22], which is approximately equivalent to 0.04 mg per mouse; therefore, this dose was used in this study. Hence the analgesic test was carried out using formulations LORF3 and LORF8 at a dose of 0.04 mg. Each dose was applied topically on the posterior paw of each mouse and the response time was recorded after 0, 30, 60, 120, and 180 min after application. The animals were observed for any gross behavioral changes, morbidity, and mortality. At time intervals from 0 to 180 min, the analgesic activity of the drug was manifested by recording the response time difference (RTD) of each mouse to lick its paws and/or jump from the hot plate. LOR injection Xefo (1.3 mg/kg ip) was used as a reference dose to compare the analgesic activity of LOR gel formulations.

### 2.10. Statistical Analysis

The data were presented as mean ± SD (*n* = 6) and statistical analysis of the cumulative amount of LOR diffused over 24 hr was carried out using Student's *t*-test. Turkey comparison test was used to compare different formulations and the level of significance was taken as *P* ≤ 0.05. The drug flux (*J*) in the steady-state region was obtained from the slope of the linear plot of the cumulative amount permeated per unit time. From the drug flux (*J*), the permeability coefficient (*K*_*p*_) was calculated using (2)

(2) $$\begin{array}{c} K_{p}=\frac{J}{\text{Co}}. \end{array}$$

The enhancement ratio (ER) was calculated from the ratio of LOR flux in the presence and absence of enhancers.

## 3. Results and Discussion

### 3.1. Solubility and Partition Coefficient

**Figure 1:**
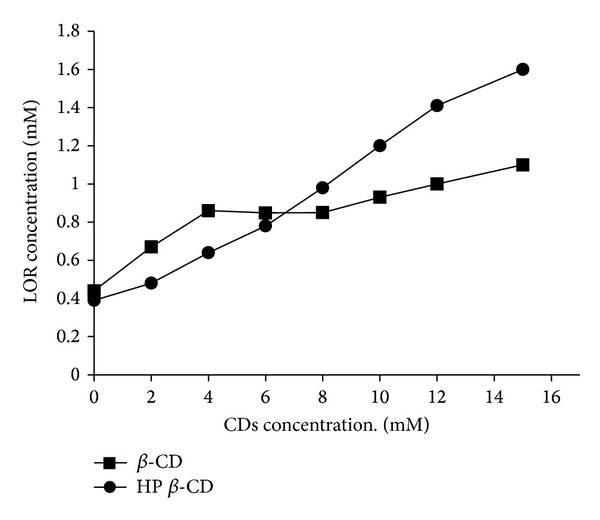
Effect of *β*-CD and HP *β*-CD concentrations on LOR solubility (in aqueous phosphate buffer, pH 7.4).

Solubility of LOR in distilled water, phosphate buffered saline (PB; pH 7.4), PB containing *β*-CD, and HP *β*-CD at 32°C was determined. The low solubility of LOR in distilled water (0.028 mg mL^−1^) was found, which proposed that LOR is lipophilic in nature. The solubility of LOR in PB (pH 7.4) was found to be 6.022 mg mL^−1^. However, LOR solubility increased linearly with the increase in the concentration of CDs (Figure 1). Regarding the effect of *β*-CD on LOR solubility, it was found that increasing *β*-CD concentrations resulted in increasing LOR solubility up to 5 mM (Figure 1). On the other hand, increasing HP *β*-CD concentrations resulted in increasing LOR solubility (Figure 1). This effect might be contributed to the formation of a stoichiometric 1 : 1 complex of LOR and HP *β*-CD [23]. This influence could be attributed to the inclusion of LOR into the HP *β*-CD cavity, thus forming the inclusion complex. The apparent 1 : 1 stability constant (*K*) of LOR with *β*-CD and HP *β*-CD was calculated as 271.6 M^−1^ and 189.5 M^−1^, respectively.

The solubility results of LOR with HP *β*-CD are comparable with the reported data, in which the solubility of LOR was enhanced by increasing HP *β*-CD concentrations in phosphate buffer solution [23].

Regarding partition coefficient, the log *p* of LOR is 1.7 and this value is less than 2.5 which means expected improvement in LOR absorption. Yano et al. [24] stated that the optimum log* p* value for NSAIDs is 2.5; so the absorption rate would increase in drugs with log *p* value less than 2.5. In the presence of 2 mM *β*-CD, partition coefficient of LOR decreased, which may be attributed to improvement in the solubility of the formed inclusion complex. Partition coefficient increased with increasing concentration of *β*-CD up to 5 mM. This increase of partition coefficient may be due to the formation of insoluble complex with *β*-CD. In case of HP *β*-CD, partition coefficient of LOR decreased with increase of the concentration of HP *β*-CD. This might be due to improvement in LOR hydrophilicity which is confirmed by the higher efficiency of the complex toward the drug than *β*-CD. It is reported that addition of methyl *β*-CD affects partition coefficient and bioavailability of certain drugs [25]. It is worth mentioning that, when transparent aqueous phase containing both *β*-CD and LOR was shacked with octanol (oil phase), precipitation occurred and was confirmed by obtaining clear aqueous phase at 2 mM of *β*-CD and turbid phase with concentration up to 5 mM. In conclusion HP *β*-CD was found to be more effective in increasing LOR solubility in phosphate buffer and more efficient in decreasing partition coefficient of LOR than *β*-CD.

### 3.2. *In Vitro* Characterization of LOR Gel Formulations

All LOR gel formulations were assessed for their cosmetic qualities such as texture, consistency, and aroma. The LOR gel formulations have a smooth texture and a pleasant and homogeneous appearance.

#### 3.2.1. pH Evaluation

The pH values of the LOR gels were found to be in the range from 7.2 to 7.8, which probably would not produce skin irritation. Hence, the prepared LOR gels are suitable for dermatological purpose.

#### 3.2.2. The Spreadability

The prepared gels should have good spreadability to meet ideal quality in the field of topical application. It is reported that the therapeutic efficiency of the gels also depends upon its spreading. The spreadability has an important character in patient compliance and helps in uniform application of the gel to the skin. If the gel has less time spread, it is considered a good gel with high spreadability value. The spreadabilty of prepared gels was of high values.

#### 3.2.3. Viscosity Measurements

**Figure 2:**
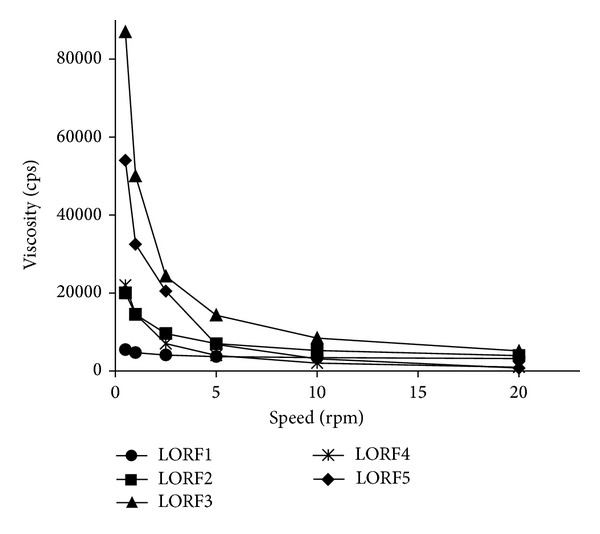
Effect of shear rate on the viscosity of different gel formulations.

Viscosity plays an important role in controlling the drug permeation. Generally viscosity of gel formulations reflects its consistency [18]. For topical analgesic formulations, the consistency of the samples is specially an important feature, due to the fact that it must be applied to the skin in thin layers. For this reason, it is preferable to formulate non-Newtonian flow system because of its low resistance to flow when applied under high shear conditions [26]. In this study, gel formulations showed non-Newtonian flow (shear thinning). As shown in Figure 2, the viscosity of the prepared LOR gels decreased with increasing shear rate values. Formulation LORF3 which contains PG had higher viscosity and LORF4 which contains ethanol showed low viscosity while LORF5 which contains a combination of PG and ethanol gave balanced viscosity value.

#### 3.2.4. Differential Scanning Calorimetric (DSC) Studies

**Figure 3:**
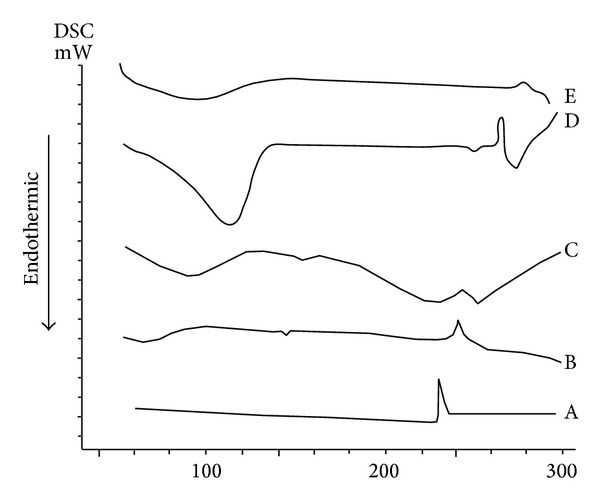
DSC thermograms of (a) LOR, (b) LOR-HPMC, (c) LOR-carpobol, (d) LOR-*β*-CD, and (e) LOR- HP *β*-CD.

DSC study has been used to detect formulation incompatibility due to interactions between drug and excipients. DSC thermograms of LOR and used excipients are shown in Figure 3. DSC thermogram of LOR showed a sharp exothermic peak at 235.2°C, which matches to its melting point indicating decomposition of LOR [27].

DSC thermogram of LOR with HPMC showed the LOR exothermic peak at 230.7°C which might be due to decomposition of LOR. Therefore, LOR is compatible with HPMC due to absence of interaction between LOR and HPMC. Moreover, DSC curve of LOR with carbopol showed an exothermic peak at 229.3°C followed by an upward line higher than the baseline of the curve which may be attributed to the fusion of the drug with decomposition. These DSC data of LOR and carbopol may be indicated to molecular dispersion of drug molecules with carbopol particles.

It is clear that the *β*-CD showed a shallow, broad endothermic peak at about 120°C which was thought to represent the vaporization of moisture from the *β*-CD sample. DSC scan of LOR and *β*-CD showed that the endothermic peak of the drug exists at the same position compared to the untreated drug, while in the case of LOR and HP *β*-CD a small exothermic peak at 230.4°C was given, which indicated LOR melting endotherm has been no longer seen. These results suggest the compatibility between LOR and the polymers.

### 3.3. *In Vitro* LOR Release

**Figure 4:**
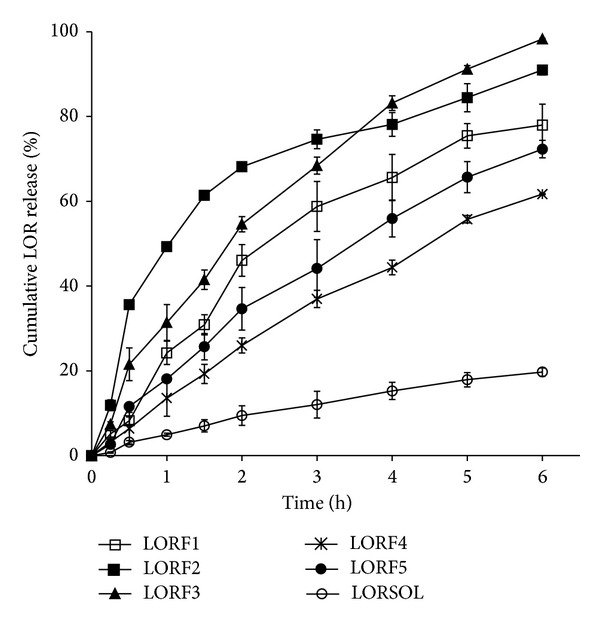
Cumulative amount of LOR released from different gel formulations. Data are presented as mean ± standard deviation (*n* = 3).

*In vitro* LOR diffused through cellophane membrane was recorded in Figure 4. It was observed that* in vitro* release data of LOR from LORF3 started slowly, that is because of its PG content which increases the viscosity of the formulation and hence lowers the release rate of the drug, but eventually the LOR release was the highest among the other formulations. PG promoted the transdermal partitions into the skin layers and thereby improved the LOR skin penetration [28]. It was reported that higher naloxone release was obtained by using PG. Different PG concentrations in water were studied by using thermal gravimetric analysis of epidermis, before and after PG addition. It was observed that 66% PG in water acts as a penetration enhancer while 100% PG extracts water from the lipid bilayer and corneocytes leading to an increase in the barrier property of SC [29]. It was also observed that 20% of PG leads to enhance permeated meloxicam from rabbit skin [30]. Ethanol is used as a cosolvent in order to increase the release of many drugs. It was known that ethanol is considered as a permeation enhancer through the skin due to disrupting the barrier structure of SC or by enhancing the solubility and partitioning of the drug in SC [31]. The maximum flux of estradiol solution for human skin was increased by increasing ethanol content up to 40–60% and was decreased with increasing ethanol above that mentioned content [31], while combination of both PG and ethanol in LORF5 exhibited negligible effect on LOR release compared to LORF2 which could be due to the fact that the dehydration effect of ethanol was blocked by humectant action of PG. According to our studies, the lowest LOR release in the drug control was observed. LORF3 gel formulation was found to be the most appropriate transdermal formulation for LOR; hence it was chosen for a comparative transdermal delivery of LOR. Regarding formulation LORF1, containing HPMC as gelling agent, it was found to have a low effect on the LOR release rate compared to LORF2 which has carbopol as gelling agent. So as a result from this experiment carbopol was found to be a good choice for formulating LOR as topical formulation and incorporation of the 20% PG even enhances the drug release rate. Depending on the obtained results (Figure 4), formulation LORF3 has been chosen to investigate the effect of different kinds of penetration enhances on LOR permeation through rabbit skin.

The penetration enhancers (5%) include Tween 80 (LORF6) and oleic acid (LORF7). Moreover, HP *β*-CD (LORF8) and *β*-CD (LORF9) have been added to LORF3 in a molar ratio of 1 : 1 of the drug (Table 2).

### 3.4. Skin Irritation Studies

Skin irritation test showed that the carbopol gel containing PG (LORF3) did not produce any irritation or sign of erythema (1.12) after being applied to volunteers. Since the skin pH is about 5.5, the prepared gels of LOR are safe, with less irritation, and suitable for dermatological purpose.

### 3.5. *Ex Vivo* Skin Permeation Studies

**Figure 5:**
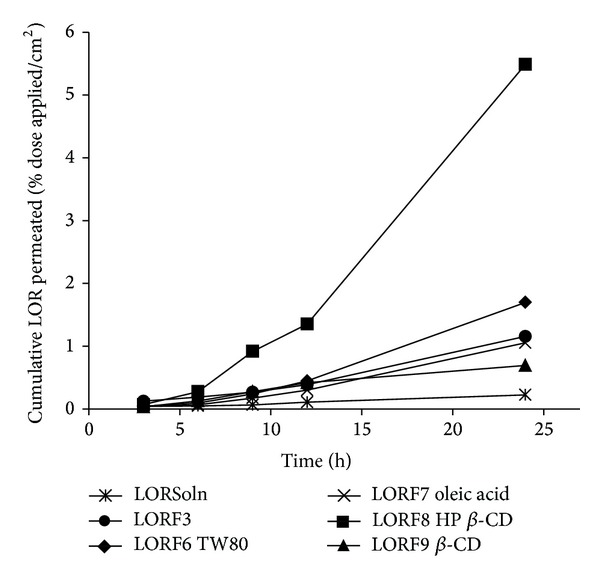
Cumulative amount of LOR permeated from carbopol gels containing different types of enhancers. Data are presented as mean ± standard deviation (*n* = 3).

The prepared LOR topical gels (LORF3) containing Tween 80 (LORF6), oleic acid (LORF7), HP *β*-CD (LORF8), and *β*-CD (LORF9) as penetration enhancers were investigated for skin permeation through excised rabbit skin [32]. The cumulative amounts of LOR that penetrated the skin over a 24-hour period from formulation are shown in Figure 5. The highest amount of LOR permeated was obtained from LORF8, while the lowest amount of permeated LOR was obtained from LORF9. The significant enhancement of LOR permeated in LORF8 contributed to the presence HP *β*-CD which may form a complex with LOR improving both its solubility and hydrophilicity as shown in the effect of HP *β*-CD on LOR solubility. On the other hand presence of *β*-CD leads to significant decrease in LOR release, which was predicted from investigating the effect of *β*-CD on the solubility and the partition coefficient of LOR as it is mentioned before.

These results were found to be in a good agreement with the data obtained by Arima [33] who showed that* in vitro* penetrated Ethyl 4-biphenyl acetate (EBA) from ointment bases was enhanced by complexation with HP *β*-CD and retarded by *β*-CD.

This enhancement was correlated with improvement in drug solubility. In the same study, the authors concluded that considerable amounts of CDs passed through skin especially HP *β*-CD. This effect may be contributed to increasing of the drug availability at the barrier surface of the skin [34]. Also, CDs are able to interact with lipid components of the SC. In another study, pure aqueous buffer solutions of *β*-CD and HP *β*-CD have been shown to be able to extract lipids from the SC [34]. It was reported that when nitroglycerine ointment complexed with dimethyl *β*-CD, it resulted in accelerating the drug permeation and retarded by complexation with *β*-CD [33]. By using HP *β*-CD, increases* in vivo* liarozol absorption were observed [35]. Surfactants are added to formulations in order to solubilize lipophilic active ingredients and so they have potential to solubilize lipids within the SC. A significant permeation of hydrocortisone and lidocaine through hairless mouse skin was observed by using Tween 80 [33].

The addition of Tween 80 to LORF3 did not give significant effect on the permeation from skin, which may be attributed to micelles formation [36]. Oleic acid (OA) is effective as penetration enhancer at relatively low concentrations (typically less than 10%) and addition of PG gives synergistic effect which promotes drug permeation from the skin [37]. The result of incorporation of 5% OA did not significantly influence the permeation of LOR compared to LORF3 which could be due to the fact that OA may induce LOR partition out of gel to SC. Also incorporation of OA may lead to increasing formulation viscosity which has the reverse effect on LOR release rate. Current investigation revealed that LOR gels have greater viscosity from oleic acid formulation than LORF3 formulation. The result is consistent with studies of EL-Megrab et al. [30] and Jantharaprapap et al. [37] who reported that meloxicam gels containing oleic acid have greater viscosity than control formulation. The reduction in LOR release rate could be attributed to the increased partition to vehicle more than skin which leads to low drug permeation. From Table 3, the flux of LOR from optimized gel (LORF8) was 14.31 ± 3.45 *μ*g cm^−2^ h^−1^ and *K*_*p*_ value of 0.00358 cm^2^ min^−1^ with ER of 18.34 (min) as compared to LORF3.

It is noteworthy that the transparency of LORF3 was kept in case of using HP *β*-CD as an enhancer while it decreased upon using the other enhancers which exhibit reduction in drug permeation through a rabbit skin.

### 3.6. *In Vivo* Analgesic Activity

Preliminary screening was carried out in six mice to study the tolerability of mice to heat by using the hot plate method. The analgesic activity of LOR after applying LORF3 or LORF8 on mice was manifested by their resistance or tolerability to the sensation of heat until licking their paws or jumping [17].  Table 4 shows the analgesic activity of LOR from LORF3 and LORF8 at doses of 0.04 each and 1.3 mg/kg ip of Xefo, a commercially available LOR injection.

**Figure 6:**
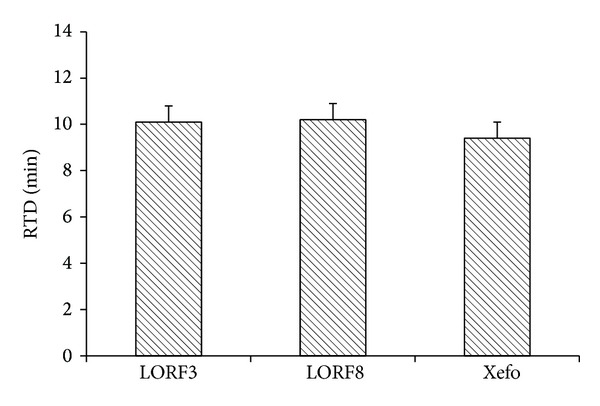
Analgesic effect of LOR gel formulation and Xefo with respect to RTD after 180 minutes.

From Table 1 and Figure 6, it could be observed that LOR gel formulations gave analgesic effect as manifested from the response time difference (RTD) compared to that of control at zero times. This indicates that analgesic action of LOR is dose dependent. In addition, since LOR has a short half-life which reaches maximum analgesic activity after 120 minutes, RTD of the gel formulations was compared to the control commercial formulation (Xefo) at 120 minutes. The analgesic studies revealed that LORF8 exhibited potent analgesic effect against thermal pain stimuli. The observed enhancement of the analgesic activity of LOR F8 and presence of oleic acid and HP *β*CD could be attributed to a number of reasons such as solubilizing and partitioning effects. It was reported that HP *β*-CD has the ability to enhance drug solubility and make it available for permeation. Alternative mechanism relied on the ability of oleic acid to decrease the integrity of the skin.

Results showed that there is no significant difference (*P* > 0.05) of the analgesic effect between LORF8 and Xefo.

## 4. Conclusion 

According to the results obtained from this study, carbopol was found to be a suitable vehicle for formulation of LOR gel. Addition of HP *β*-CD to LOR enhanced its solubility in phosphate buffer solution and lowered its partition coefficient. The incorporation of HP *β*-CD as penetration enhancer to carbopol gel formulation improved LOR permeability through rabbit skin. The analgesic activity of LOR gel formulations (LORF3 and LORF8) in mice showed similar effect compared to LOR commercial product (Xefo). This study revealed that the carbopol gel formulation containing PG and HP *β*-CD could be a promising carrier for topical delivery of LOR.

## Figures and Tables

**Table 1:** Composition of LOR gel formulations.

Materials	Formulations composition (% w/w)				
	LORF1	LORF2	LORF3	LORF4	LORF5
LOR	0.8	0.8	0.8	0.8	0.8
HPMC	15	—	—	—	—
Carbopol 974	—	1	1	1	1
PG	—	—	20	—	20
Ethanol	—	—	—	40	40
Methylparaben	0.2	0.2	0.2	0.2	0.2
Triethanolamine	2	2	2	2	2
Water to	100	100	100	100	100

**Table 2:** Composition of carbopol gel formulations containing different enhancers.

Materials	Formulations composition (% w/w)				
	LORF3	LORF6	LORF7	LORF8	LORF9
LOR	0.8	0.8	0.8	0.8	0.8
Carbopol 974	1	1	1	1	1
PG	20	20	20	20	20
Tween 80	—	5	—	5	5
Oleic acid	—	—	5	5	5
HP *β*-CD∗	—	—	—	5	—
*β*-CD∗	—	—	—	—	5
Methylparaben	0.2	0.2	0.2	0.2	0.2
Triethanolamine	2	2	2	2	2
Water to	100	100	100	100	100

*LOR : CDs (1 : 1).

**Table 3:** Percutaneous permeability parameters of LOR formulations through rabbit skin.

Formulations	ER	J (*μ*g cm^−2^ h^−1^)	*K*_*p*_ (cm h^−1^) (×10^−5^)
LORF3	—	0.78 ± 0.21	1.8
LORF6	1.83	1.43 ± 0.47	36
LORF7	1.69	1.32 ± 0.51	33
LORF8	18.34	14.31 ± 3.45	358
LORF9	1.18	0.92 ± 0.22	23

**Table 4:** Analgesic activity of LOR formulations at a dose of 0.04 mg compared to injectable LOR dosage form (Xefo) on mice.

Time	Formulations					
	LORF3	RTD	LORF8	RTD	Xefo	RTD
Initial	6.3 ± 1.3	0	6.9 ± 0.5	0	6.7 ± 0.7	0
30	8.0 ± 1.5	1.7	8.7 ± 1.3	2.2	8.5 ± 1.1	1.8
60	8.4 ± 1.1	2.1	8.4 ± 0.7	2.7	9.0 ± 1.1	2.2
120	10.1 ± 2	3.8	10.2 ± 2.7	4.3	8.4 ± 0.8	1.7
180	8.7 ± 0.8	2.3	10.3 ± 1.2	4	8.6 ± 0.9	1.5

## References

[1] Hanada S, Fujioka K, Futamura Y, Manabe N, Hoshino A, Yamamoto K (2013). Evaluation of anti-inflammatory drug-conjugated silicon quantum dots: their cytotoxicity and biological effect. *International Journal of Molecular Sciences*.

[2] Whitehouse MW (2011). Anti-inflammatory glucocorticoid drugs: reflections after 60 years. *Inflammopharmacology*.

[3] Cevc G, Blume G (2001). New, highly efficient formulation of diclofenac for the topical, transdermal administration in ultradeformable drug carriers, Transfersomes. *Biochimica et Biophysica Acta. Biomembranes*.

[4] Mathy F-X, Ntivunwa D, Verbeeck RK, Préat V (2005). Fluconazole distribution in rat dermis following intravenous and topical application: a microdialysis study. *Journal of Pharmaceutical Sciences*.

[5] Karadzovska D, Brooks JD, Riviere JE (2013). Modeling the effect of experimental variables on the in vitro permeation of six model compounds across porcine skin. *International Journal of Pharmaceutics*.

[6] Herrmann WA, Geertsen MS (2009). Efficacy and safety of lornoxicam compared with placebo and diclofenac in acute sciatica/lumbo-sciatica: an analysis from a randomised, double-blind, multicentre, parallel-group study. *International Journal of Clinical Practice*.

[7] Ammar HO, Ghorab M, Mahmoud AA, Makram TS, Noshi SH (2012). Topical liquid crystalline gel containing lornoxicam/cyclodextrin complex. *Journal of Inclusion Phenomena and Macrocyclic Chemistry*.

[8] Shahzad Y, Khan Q, Hussain T, Shah SNH (2013). Influence of cellulose derivative and ethylene glycol on optimization of lornoxicam transdermal formulation. *International Journal of Biological Macromolecules*.

[9] Yener G, Üner M, Gönüllü U (2010). Design of meloxicam and lornoxicam transdermal patches: preparation, physical characterization, ex vivo and in vivo studies. *Chemical and Pharmaceutical Bulletin*.

[10] Kavitha K, Rajendra MM (2011). Design and evaluation of transdermal films of lornoxicam. *International Journal of Pharma and Biosciences*.

[11] Valenta C, Auner BG (2004). The use of polymers for dermal and transdermal delivery. *European Journal of Pharmaceutics and Biopharmaceutics*.

[12] Iervolino M, Cappello B, Raghavan SL, Hadgraft J (2001). Penetration enhancement of ibuprofen from supersaturated solutions through human skin. *International Journal of Pharmaceutics*.

[13] Sun JD, Frantz SW, Beskitt JL (1995). In vitro skin penetration of ethylene glycol using excised skin from mice and humans. *Journal of Toxicology. Cutaneous and Ocular Toxicology*.

[14] Chawla V, Saraf SA (2012). Rheological studies on solid lipid nanoparticle based carbopol gels of aceclofenac. *Colloids and Surfaces B: Biointerfaces*.

[15] Carnali JO, Naser MS (1992). The use of dilute solution viscometry to characterize the network properties of carbopol microgels. *Colloid & Polymer Science*.

[16] Lane ME (2013). Skin penetration enhancers. *International Journal of Pharmaceutics*.

[17] Alomrani A (2002). *Effect of formulation, chemical enhancer and ultrasound on the percuteous absorption of isradipine through rabbit skin [M.S. thesis]*.

[18] Shinde U, Pokharkar S, Modani S (2012). Design and evaluation of microemulsion gel system of nadifloxacin. *Indian Journal of Pharmaceutical Sciences*.

[19] Shetty SN, Anika SM (1982). *Laboratory Manual of Pharmacology and Toxicology*.

[20] Franzotti EM, Santos CVF, Rodrigues HMSL, Mourão RHV, Andrade MR, Antoniolli AR (2000). Anti-inflammatory, analgesic activity and acute toxicity of Sida cordifolia L. (Malva-branca). *Journal of Ethnopharmacology*.

[21] Medhi B, Khanikor HN, Lahon LC, Mohan P, Barua CC (2003). Analgesic, anti-inflammatory and local anaesthetic activity of Moringa pterygosperma in laboratory animals. *Pharmaceutical Biology*.

[22] Bianchi M, Panerai AE (2002). Effects of lornoxicam, piroxicam, and meloxicam in a model of thermal hindpaw hyperalgesia induced by formalin injection in rat tail. *Pharmacological Research*.

[23] Hamza YE-S, Aburahma MH (2009). Design and in vitro evaluation of novel sustained-release double-layer tablets of lornoxicam: utility of cyclodextrin and xanthan gum combination. *AAPS PharmSciTech*.

[24] Yano T, Nakagawa A, Tsuji M, Noda K (1986). Skin permeability of variousnon-steroidal anti-inflammatory drugs in man. *Life Sciences*.

[25] Nakai Y, Yamamoto K, Terada K, Horibe H (1984). Interaction of tri-O-methyl-*β*-cyclodextrin with drugs. *Journal of Inclusion Phenomena*.

[26] Bousmina M (1999). Rheology of polymer blends: linear model for viscoelastic emulsions. *Rheologica Acta*.

[27] O'Neil MJ, Heckelman PE, Koch CB, Roman KJ (2006). An encyclopedia of chemicals, drugs, and biologicals. *The Merck Index*.

[28] Ho H-O, Huang F-C, Sokoloski TD, Sheu M-T (1994). The influence of cosolvents on the in-vitro percutaneous penetration of diclofenac sodium from a gel system. *Journal of Pharmacy and Pharmacology*.

[29] Panchagnula R, Bokalial R, Sharma P, Khandavilli S (2005). Transdermal delivery of naloxone: skin permeation, pharmacokinetic, irritancy and stability studies. *International Journal of Pharmaceutics*.

[30] El-Megrab NA, El-Nahas HM, Balata GF (2006). Formulation and evaluation of meloxicam gels for topical administration. *Saudi Pharmaceutical Journal*.

[31] Obata Y, Takayama K, Maitani Y, Machida Y, Nagai T (1993). Effect of ethanol on skin permeation of nonionized and ionized diclofenac. *International Journal of Pharmaceutics*.

[32] Bronough R, Maibach HI (1985). *Percutaneous Absorption*.

[33] Arima H, Adachi H, Irie T, Uekama K, Pitha J (1990). Enhancement of the antiinflammatory effect of ethyl 4-biphenyl acetate in ointment by *β*-cyclodextrin derivatives: increased absorption and localized activation of the prodrug in rats. *Pharmaceutical Research*.

[34] Loftsson T, Masson M (2001). Cyclodextrins in topical drug formulations: theory and practice. *International Journal of Pharmaceutics*.

[35] Bentley MV, Vianna RF, Wilson S, Collett JH (1997). Characterization of the influence of some cyclodextrins on the stratum corneum from the hairless mouse. *Journal of Pharmacy and Pharmacology*.

[36] Sarpotdar PP, Zatz JL (1986). Percutaneous absorption enhancement by nonionic surfactants. *Drug Development and Industrial Pharmacy*.

[37] Jantharaprapap R, Stagni G (2007). Effects of penetration enhancers on in vitro permeability of meloxicam gels. *International Journal of Pharmaceutics*.

